# Euglycemic Diabetic Ketoacidosis in the ICU: 3 Case Reports and Review of Literature

**DOI:** 10.1155/2018/1747850

**Published:** 2018-10-01

**Authors:** Pablo Lucero, Sebastián Chapela

**Affiliations:** ^1^Hospital Británico de Buenos Aires, Intensive Care Services, Argentina; ^2^Universidad de Buenos Aires, Facultad de Medicina, Departamento de Bioquimica Humana, Argentina

## Abstract

Diabetic ketoacidosis (DKA) is an acute complication of diabetes mellitus, both type I and type II, as well as other types with diabetes such gestacional diabetes mellitus. It is characterized by blood glucose levels greater than 250 mg/dL and metabolic acidosis (pH < 7.3 and serum bicarbonate < 15 mEq/dL) with an increased anion gap and the presence of ketone bodies in the blood or urine. Within this pathology, there is a subgroup of pathologies which are characterized by being present with no signs of hyperglycemia, posing a diagnostic challenge due to the absence of the main sign of the pathology and the diversity of their pathophysiology. In this article, we will present 3 clinical cases with 3 different forms of clinical presentation: a case of DKA in pregnancy, a case of DKA associated with the use of sodium-glucose cotransporter 2 (SGLT-2) inhibitors, and a third case related to sepsis, together with a narrative review of the literature on the topic.

## 1. Introduction

Diabetic ketoacidosis is an acute complication of diabetes. It is diagnosed through laboratory results showing metabolic acidosis with an increased gap and evidence of ketone bodies in the blood or urine. Most of the time, it is present with hyperglycemia. The clinical presentation of this pathology is diverse, going from abdominal pain to sensory deterioration and coma [1].

The pathophysiology of hyperglycemia in diabetic ketoacidosis has 3 cornerstones: an increase in gluconeogenesis, an increase in glycogenolysis and a decrease in peripheral glucose uptake due to a decrease in insulin action in the receptors or a decrease in insulin levels [1]. This prevents glucose from being transported inside the cells and being used as metabolic fuel. On the other hand, there is an increase in lipolysis and fatty acids start being used in the liver, where they are metabolized into ketone bodies, which can be absorbed by most cells [1].

Diabetic ketoacidosis is defined by the presence of blood glucose levels greater than 250 mg/dL, being this the main finding, associated with metabolic acidosis (pH < 7.3 and serum bicarbonate < 15 mEq/dL) with an increased anion gap and the presence of ketone bodies in the blood and/or urine [1]. There are different forms of presentation which differ from the usual presentation described in literature, such as the case of normoglycemic diabetic ketoacidosis. This pathology was first described by Munro in 1973 [2] but, in his work, he studied patients with blood glucose levels under 300mg/dL. Currently, the definition is in line with blood glucose levels under 250mg/dL [1]. 6% of patients show blood glucose levels under 300 mg/dL and around 1% of patients show levels under 180 mg/dL. The most common causes are insulin administration on the way to the hospital and fasting [1]. The diagnosis and treatment of this pathology require a deep pathophysiological knowledge, since it can be triggered by different etiologies. In this review, we will present 3 completely different cases of normoglycemic diabetic ketoacidosis.

## 2. Clinical Case 1

A 22-year-old woman with a history of diabetes mellitus (diagnosed at 7 years old) is treated with insulin glargine and with good adherence to treatment, with hypothyroidism and 2 previous ICU admissions due to diabetic ketoacidosis in which blood glucose levels were greater than 300 mg/dL.

The patient sought consultation due to vomiting and abdominal pain 12 hours after onset. Upon physical examination, the abdomen was distended with diffuse pain and no signs of peritoneal irritation. Laboratory results showed the following values: pH: 7.25; bicarbonate: 10 mEq/dL; BE: -14.9; blood glucose: 153 mg/dL and positive ketonemia. Admission laboratory results are shown in Table 1. Upon diagnosis of normoglycemic diabetic ketoacidosis, in the context of menstrual cycle alterations and with the aim of studying the trigger, beta subunit of human chorionic gonadotropin levels was requested: 98.928 IU/L. A transvaginal ultrasound was performed and showed a gestational sac with an embryo inside. Reanimation was started with parenteral crystalloids administered at 250 mL/h during 24 hrs. It was interspersed isotonic saline solutions and polyelectrolyte solutions. Total income is 7000 ml / 24 hs. Urinary volume is 2750 ml / 24 hs. Positive balance is 4250 ml/24 hs. Continuous insulin infusion was started, as described in literature (receiving a total of 100 IU in 48 hrs). Progress was shown with improvement of the clinical condition and lab monitoring every 8 hours: pH 7.47; bicarbonate of 22 mEq/dL with blood glucose levels in the normal range (< 200 mg/dl). The usual insulin glargine dose was restored and the patient was discharged.

## 3. Clinical Case 2

A 50-year-old woman, former smoker, with a history of arterial hypertension, dyslipidemia, left side breast cancer which required chemotherapy, radiation therapy and surgery, hypothyroidism, and diabetes mellitus type II, is treated with 10 mg/day of Dapagliflozin, 1000 mg of Metformin every 12 hours, and NPH insulin at 40 and 60 IU. The patient sought consultation due to abdominal pain, diarrhea and fever. Upon admission, the patient was alert, tachypneic, and being with diffuse abdominal pain with no sign of peritoneal irritation. An abdominal ultrasound was requested and showed the gallbladder with multiple gallstones. The complete laboratory results are shown in Table 1. In the context of leukocytosis, acute kidney failure, and severe metabolic acidosis, the patient was admitted to the ICU with a diagnosis of sepsis. Due to the presence of metabolic acidosis with a gap of 32, a ketonemia test was requested. The result was positive and the patient was diagnosed with euglycemic diabetic ketoacidosis.

After starting treatment with a continuous insulin infusion pump and the administration of water, the patient was discharged from the hospital after 5 days.

## 4. Clinical Case 3

A 74-year-old male patient with a history of arterial hypertension, noninsulin dependent diabetes mellitus medicated with oral hypoglycemic agents, ischemic cardiopathology with stent placement, nonoliguric chronic kidney failure, and cryptogenic liver cirrhosis required a liver transplant and subsequently suffered portal vein thrombosis requiring anticoagulation. The patient sought consultation after 3 days of passing liquid stools, together with emesis. He denied having fever spikes and, on that date, consulted the emergency ward of this institution, to which he was admitted feeling alert, with AT: 130/64, heart rate: 108 beats per minute, and SO2: 97% on room air. Upon physical examination, the patient was alert, tachypneic, and being with dry mucous membranes. Admission laboratory results are shown in Table 1. A ketonemia test was requested and the result was positive. The clinical presentation was interpreted as dehydration secondary to gastrointestinal losses and euglycemic diabetic ketoacidosis. Reanimation was started with crystalloids, a continuous insulin infusion pump, and the administration of intravenous bicarbonate. After 48 hrs, the patient presented DKA resolution criteria.

## 5. Discussion

Euglycemic diabetic ketoacidosis is a diagnostic challenge for treating physicians, since there is no hyperglycemia. On the other hand, there are many causes of metabolic acidosis in patients in the intensive care unit, although, when analyzing the gap, high gap metabolic acidosis is less frequent than hyperchloremic acidosis [14]. Therefore, knowing this pathology is key when treating patients with diabetes. Moreover, the triggers are varied and, in this study, we presented 3 cases with two different pathophysiological causes.

This pathology is triggered by multiple causes (Table 2). The following pathophysiological mechanisms are common to all causes: a decrease in insulin action or secretion with a decrease in total glucose uptake at a cellular level, an increase in the production of counterregulatory hormones, and a decrease in glucose production by the liver or an increase in the excretion of glucose in the urine [11, 12].

The first case deals with a diabetic patient who is pregnant. The reason that normal pregnancy increases blood glucose levels is based on the progressive insulin resistance, which normally occurs. This resistance also explains the worsening of pregestational diabetes during pregnancy. The exogenous insulin loses its effect as the pregnancy progresses. These effects are attributable to the destruction of insulin by the kidney and the action of placental insulinases.

At the beginning of pregnancy, insulin maintains its activity, and its concentration increases due to the hyperplasia of the Beta cells of the pancreatic islets, induced by the high concentrations of placental steroids. As a result of these changes, fasting glycemia decreases. The main effect of insulin in the body is to allow the storage of nutritious substrates to meet energy needs. The provision of food is intermittent while the consumption of energy is constant from where the need for storage arises. The maternal organism stores energies in the form of glucose and fats. In addition, human chorionic gonadotropin causes vomiting, which causes fasting, dehydration, and metabolic acidosis [15].

As pregnancy progresses, the activity of the usual counterregulatory hormones such as human placental lactogen, which is synthesized by the trophoblast and released into the circulation, reduces maternal sensitivity to insulin, increasing postprandial blood glucose levels [10]. Progesterone reduces gastrointestinal motility, increasing glucose uptake [10]. In addition, there is a decrease in insulin sensitivity, particularly in the third trimester, caused by hormonal changes that occur during pregnancy like an increase in estrogen, progestogens, human placental lactogen, and secretion of TNF-*α* [15]. All these mechanisms induce hyperglycemia in pregnancy. On the other hand, the placenta and the fetus absorb large amounts of glucose, decreasing blood levels when fasting. This leads to an increase in the secretion of maternal fatty acids and their subsequent metabolization in ketone bodies [12].

During late pregnancy, the fetus dramatically increases its glucose-based metabolism and accentuates its anabolic process by growth. On the other hand, the maternal metabolism enters a catabolic process in order to send all the glucose to the fetus through the placenta, using fat as the primary fuel. In the diabetic patient, the decrease in insulin intake profoundly affects the general metabolism, particularly at the level of liver, muscle, and adipose tissue, which are insulin essential action points. The absence of this hormone causes distortion of homeostasis. Plasma levels of glucose, free fatty acids and ketones rise to extreme figures, plasma pH and bicarbonate fall dangerously and there is marked loss of fatty tissue and body mass. If insulin levels are not restored, this case can lead to death.

Finally, the respiratory alkalosis that occurs during pregnancy increases the urinary excretion of bicarbonate, reducing the ability to buffer pH changes caused by the increase in body ketone production [16]. This leads to euglycemic diabetic ketoacidosis in pregnancy.

The incidence rate of diabetic ketoacidosis in all pregnant women with diabetes varies between 0.5 and 3%, being more common in patients with type I diabetes. However, there are more and more cases of patients with type II and gestational diabetes [17, 18]. In a unicentric study in which 223,000 deliveries were analyzed, 14,532 (6.5%) were complicated due to diabetes, just 33 patients presented 40 diabetic ketoacidosis episodes with average blood glucose levels of 380 mg/dL on admission, whereas only 3 cases presented euglycemic diabetic ketoacidosis [18]. The different cases of euglycemic diabetic ketoacidosis in pregnancy, their initial diagnosis, and clinical presentations are analyzed in Table 3. In contrast to most of the cases described in literature, our patient presented with DKA during the first trimester.

The harmful effects of ketoacidosis on the fetus are caused by ketone bodies and glucose passing the placental barrier, dehydration, which leads to decreased placental perfusion and electrolyte imbalance [18]. Fetal acidosis is caused by hyperglycemia, which leads to osmotic diuresis and fetal intravascular volume depletion. Fetal hyperinsulinemia increases oxygen uptake. A decrease in 2,3-DPG increases oxygen affinity for hemoglobin, reducing the amount of oxygen available to the fetus and generating hypoxia [17]. The electrolyte disturbance can not only generate maternal arrhythmias with a subsequent decrease in placental perfusion, but also generate fetal arrhythmias and risk of cardiorespiratory arrest [18]. Although there are no studies that show the long-term consequences for the fetuses born alive, neurodevelopmental alterations were observed. In contrast to other pregnancy complications, a hasty delivery with DKA would be harmful to the fetus. Therefore, it is recommended to stabilize the mother first [19]. Some studies state that fetal mortality in patients with DKA can reach 9% [15] and perinatal mortality is between 9 and 35% [17]. However, there are also authors who argue that ketoacidosis is not associated with a higher mortality rate during the first trimester, nor with a higher rate of malformations [20].

The mainstay of treatment does not differ from the treatment for hyperglycemic ketoacidosis, that is, hydration and insulin. The difference is that, in order to maintain blood glucose levels, the amount of glucose administered must be higher and, in the case of pregnant patients, care should be taken to maintain blood glucose levels suitable for fetal welfare. There is evidence in literature showing that a value of 250 mg/dL (Baha M. 2014) or values between 100 and 150 mg/dL would accomplish this [20].

The second case is associated with the use of sodium-glucose cotransporter 2 (SGLT-2) inhibitors. The incidence rate of diabetic ketoacidosis in patients treated with SGLT-2 inhibitors varies between 0.16 and 0.76 cases per 1000 patients per year [21, 22]. In a review of literature, 46 cases of diabetic ketoacidosis associated with the use of SGLT-2 were found and, in 70% of the cases, the ketoacidosis was euglycemic [23]. The main mechanism of action is the inhibition of glucose uptake in proximal tubules, increasing glycosuria [24]. In addition, SGLT-2 inhibitors significantly increase plasma glucagon levels through a decrease in paracrine inhibition of insulin and possibly due to the inhibition of glucose transport into pancreatic *α* cells by SGLT-2 [22]. At the same time, they decrease 3-hydroxybutyrate and acetoacetate elimination at the kidney level [24–28]. Moreover, when blood glucose levels decrease, patients that are being treated with insulin decrease its administration. Therefore, counterregulatory hormone effects predominate, resulting in a lower inhibition of lipolysis and lipogenesis and, thereby, triggering euglycemic ketoacidosis [29–31]. Case reports include the 3 drugs of the gliflozin class: Dapagliflozin [24, 25, 29–35], Canagliflozin [26–28, 31–33, 36, 37], and Empagliflozin [38–40].

The last case deals with a patient with diabetic ketoacidosis associated with dehydration. During fasting, when hepatic glycogen is consumed, there is no source of glucose release into the bloodstream; however, lipolysis and the generation of ketone bodies are increased [41]. Dehydration is also a factor that contributes to the development of euglycemia [42].

Luethi et al. [43] analyzed blood glucose levels, arterial blood gases, and ketonemia and ketonuria in 60 critically ill patients. 63% of the patients developed some degree of ketosis (*β*-hydroxybutyric levels greater than 0.6 mmol/L). In 12% of the patients, it was severe (greater than 3 mmol/L), and 33 % developed ketonuria (which was only severe in 2% of the patients). The prevalence of ketosis was the same in those who presented glucose peaks greater than 180 mg/dL and those who did not [1]. It is interesting to observe that, in this study [44], only 2 patients out of the 60 developed ketoacidosis based on the criteria set forth by the Joint British Diabetes Society [45] and none of them did, based on the ADA's criteria [11].

Finally, another possible cause of euglycemic ketoacidosis is the administration of insulin before being admitted to the hospital [42]. Other causes are pancreatic lesions developed during pancreatitis due to alcohol consumption, associated with the fasting required by this condition, which would explain the development of euglycemic ketoacidosis [42]. Furthermore, cocaine abuse causes an increase in the secretion of cortisol and noradrenaline by the adrenal gland, in addition to the anorexigenic effects of this drug, which lead to fasting [46].

## 6. Conclusion

Euglycemic diabetic ketoacidosis is a diagnostic challenge, not only due to the absence of its most important sign, which is hyperglycemia, but also due to its varied triggers. Knowing the different contexts in which it can occur will allow us to suspect euglycemic diabetic ketoacidosis and begin rapid and adequate treatment of the precipitating cause, as well as aggressive hydration, glucose homeostasis through insulin administration, and the adjustment of electrolyte imbalances. A delay results in serious complications both in the fetus (in the case of gestational diabetes) and in the patient, increasing in-hospital morbidity and mortality.

## Figures and Tables

**Table 1:** Patients' laboratory results upon admission to the ICU.

	**Na+**	**K+**	**Cl**	**pH**	**HCO2**	**BE**	**GAP**	**Δ CL**	**Ketonemia**	**Blood Glucose**
	**(mEq/L)**	**(mEq/L)**	**(mEq/L)**		**(mEq/L)**	**(mEq/L)**				
										**(mg/dL)**
***CASE 1***	137	4.9	102	7.25	10	-14.9	23	-0.75	+	153

***CASE 2***	142	3.9	108	7.13	2	-23.7	32	1.5	+	165

***CASE 3***	140	4.8	109	7.28	7.3	-16.8	23	4	+	132

**Table 2:** Causes of euglycemic diabetic ketoacidosis.

Fasting
Insulin use prior to hospital admission
Pregnancy
Use of SGLT-2
Cocaine abuse
Pancreatitis
Cirrhosis
Use of insulin pump
Sepsis

SGLT-2: type 2 sodium-glucose cotransporter.

**Table 3:** Cases of euglycemic DKA in pregnancy reported in literature.

**Author and Year**	**Age of the Patient**	**Gestational Week**	**Obstetric History**	**History of Diabetes**	**Blood Glucose upon Admission**	**pH upon Admission**	**Triggering Factor**
Darhambulla, 2012 [3]	30 years old	33	Pregnancy: 2 Borns: ?	Recently diagnosed gestational diabetes	95 mg/dL	7.17	Urinary tract infection

Cardonell, 2016 [4]	33 years old	35	Pregnancy: 3 Borns: 2	Diabetes type II	134 mg/dL	7.02	Unknown. During delivery

Chico, 2008 [5]	29 years old	24	Pregnancy: 2 Borns: 1	Diabetes type I	93 mg/dL	7.22	Unknown. During delivery

Franke, 2001 [6]	23 years old	32	Unknown	Recently diagnosed gestational diabetes	127 mg/dL	7.2	Influenza A infection

Kamalakannan, 2003 [7]	28 years old	36	Pregnancy: 5 Borns:?	Diabetes type I	234 mg/dL	7.1	Vomiting and poor treatment adherence

Karpate, 2013 [8]	25 years old	37	Pregnancy: 1 Borns: 0	None	102 mg/dL	N/A	Fasting

Napoli, 2011 [9]	26 years old	34	Pregnancy: 3 Borns: 2	Diabetes type I	211 mg/dL	7.25	Altered food intake

Oliver, 2007 [10]	29 years old	28	N/A	Diabetes type I	205 mg/dL	7.15	Bronchial pneumonia

Rivas, 2016 [11, 12]	39 years old	32	Pregnancy: 2 Borns: 1	None	225 mg/dL	7.15	Emesis

Tarif, 2007 [13]	37 years old	35	Pregnancy: 5 Borns: 4	Gestational diabetes	78 mg/dL	7.32	Emesis and diarrhea
